# 3D vena contracta area after MitraClip© procedure: precise quantification of residual mitral regurgitation and identification of prognostic information

**DOI:** 10.1186/s12947-017-0120-9

**Published:** 2018-01-09

**Authors:** Alexander Dietl, Christine Prieschenk, Franziska Eckert, Christoph Birner, Andreas Luchner, Lars S. Maier, Stefan Buchner

**Affiliations:** 1 Department of Internal Medicine II, University Hospital Regensburg, Franz-Josef-Strauss Allee 11, D-93053 Regensburg, Germany; 2 Comprehensive Heart Failure Center Würzburg, University Hospital and University of Würzburg, Würzburg, Germany; 3 Department of Internal Medicine I, Klinikum St. Marien, Amberg, Germany; 4 Department of Internal Medicine II, Sana Kliniken Cham, Cham, Germany

**Keywords:** Percutaneous mitral valve repair, MitraClip, 3D echocardiography, Vena contracta area, Six-minute walk test, NT-proBNP, Prognosis, Functional mitral regurgitation

## Abstract

**Background:**

Percutaneous mitral valve repair (PMVR) is increasingly performed in patients with severe mitral regurgitation (MR). Post-procedural MR grading is challenging and an unsettled issue. We hypothesised that the direct planimetry of vena contracta area (VCA) by 3D–transoesophageal echocardiography allows quantifying post-procedural MR and implies further prognostic relevance missed by the usual ordinal scale (grade I-IV).

**Methods:**

Based on a single-centre PMVR registry containing 102 patients, the association of VCA reduction and patients’ functional capacity measured as six-minute walk distance (6 MW) was evaluated. 3D–colour-Doppler datasets were available before, during and 4 weeks after PMVR.

**Results:**

Twenty nine patients (age 77.0 ± 5.8 years) with advanced heart failure (75.9% NYHA III/IV) and severe degenerative (34%) or functional (66%) MR were eligible. VCA was reduced in all patients by PMVR (0.99 ± 0.46 cm^2^ vs. 0.22 ± 0.15 cm^2^, *p* < 0.0001). It remained stable after median time of 33 days (*p* = 0.999). 6 MW improved after the procedure (257.5 ± 82.5 m vs. 295.7 ± 96.3 m, *p* < 0.01). Patients with a decrease in VCA less than the median VCA reduction showed a more distinct improvement in 6 MW than patients with better technical result (*p* < 0.05). This paradoxical finding was driven by inferior results in very large functional MR.

**Conclusions:**

VCA improves the evaluation of small residual MR. Its post-procedural values remain stable during a short-term follow-up and imply prognostic information for the patients’ physical improvement. VCA might contribute to a more substantiated estimation of treatment success in the heterogeneous functional MR group.

**Electronic supplementary material:**

The online version of this article (10.1186/s12947-017-0120-9) contains supplementary material, which is available to authorized users.

## Background

Percutaneous mitral valve repair (PMVR) by the MitraClip**©**-system (Abbott Vascular) has evolved as successful alternative to surgery for the treatment of severe mitral regurgitation (MR) in patients at high surgical risk [1]. Due to edge-to-edge technique at least two neo-orifices are created by the procedure. Therefore, established parameters of grading MR recommended by current guidelines like width of vena contracta and effective regurgitant orifice area [2–4] are not appropriate for the complex post-procedural mitral valve anatomy. The few existing recommendations for MR grading after PMVR get by with a multimodal approach integrating parameters as visual assessment of regurgitant jet [5], which are semi-quantitative and subjectively influenced. However, vena contracta area (VCA) cannot only be approximated by the PISA method, but also be directly measured by cardiac magnetic resonance imaging [6, 7] or three-dimensional transoesophageal echocardiography (3D–TEE) [8]. As 3D–TEE was known to be reliable in multiple VCA [9], its use for the MR assessment after PMVR appeared reasonable. Recently, the feasibility of direct VCA measurement in multiple neo-orifices was demonstrated with a significant decrease of VCA by PMVR [10, 11]. Post-procedural VCA is supposed to be more precise than an ordinal scaled MR grading - as if the imaging resolution in grade I and II MR is increased. However, this incremental parameter will serve little purpose, unless it implies any prognostic information. To date, data on the prognostic relevance of VCA reduction for the patients’ functional outcome after PMVR is lacking. Therefore, we analysed the data of a single-centre registry containing 102 patients, who underwent PMVR, in order to examine the association of VCA-reduction and patients’ functional capacity measured as six-minute walking distance.

## Methods

### Study population

The PMVR registry of the University Hospital Regensburg, Germany, comprises 102 patients, who underwent the procedure between 04/2012 and 12/2015 and were screened for eligibility. Inclusion criteria for this study were a standardised six-minute walk test before and after PMVR as well as stored 3D–TEE colour Doppler datasets before and during PMVR. For this investigation, we excluded 73 patients (unavailable six-minute walk test, 48 subjects; unavailable 3D–TEE colour Doppler dataset, 17 subjects; insufficient quality of stored echocardiography for VCA determination, 8 subjects), yielding 29 cases for this analysis.

### Clinical parameters

Further information concerning the patients’ health status was derived from medical records. EuroScoreII and logEuroScore were calculated [12]. A six-minute walk test (6MW) was recorded before PMVR and 4 weeks after the procedure. The test was performed according to the current statement of the American Thoracic Society [13] indoors, along a flat, straight, enclosed, seldom travelled corridor with a hard surface by a trained nurse. The percutaneous-repair procedure was performed under general anaesthesia with the MitraClip System (Abbott Vascular, Lake Bluff, USA) as previously described [1, 14].

### Echocardiography

Two-dimensional transthoracic echocardiography (iE-33 ultrasound system with S5–1 transducer; Philips Medical Systems, Amsterdam, The Netherlands) was performed in all patients before and 4 weeks after PMVR. Left ventricular volumes and left ventricular ejection fraction were calculated by Simpson’s rule according to recent guidelines [15]. MR was quantified in an integrative view according to the Endovascular Valve Edge-to-Edge REpair STudy (EVEREST) criteria [16]. Information on valve morphology, colour flow doppler, presence or absence of systolic pulmonary vein flow, regurgitant volume and regurgitant fraction was gathered according to recent guidelines [2–4] and combined to grade MR on a scale from mild to severe (I to IV) [16] to assure comparability to previously published registries [1, 17–21]. MR immediately after PMVR was graded from I to IV according to the recommendations of the German Cardiac Society (DGK, Additional file 1).

All patients underwent TEE for screening (“before PMVR”) purpose and during the catheter intervention providing a dataset immediately after Clip release (“immediately after PMVR”). In a subgroup, a follow-up TEE was performed 4 weeks after PMVR (“Follow up”). All images were acquired using an iE-33 ultrasound system equipped with a 3D–matrix array transducer (X7-2 t). Screening and follow-up examinations were done in conscious sedation using benzodiazepines. General anaesthesia was established for PMVR. The aetiology of mitral regurgitation was described as degenerative (DMR) or functional (FMR).

**Fig. 1:**
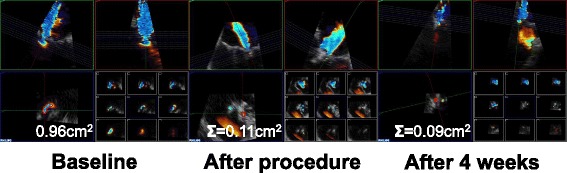
Vena contracta area determination by 3D–TEE is exemplarily shown for a DMR patient. Shown are MPR views at three time points. Each MPR view is based on one recorded data set and composed of a fourfold table. Top left (green box): midoesophageal long axis view. Top right (red): orthogonal plane to green box. Bottom left (blue): 3D–en face view to VCA (traced by red line). Bottom right: multislice representation of 9 evenly distributed slices parallel to 3D–en face view, used to find actual VCA plane. VCA was defined as the central laminar jet core (within red lines) as defined by current ASE guidelines [2]. Immediately after PMVR the residual regurgitant jet was split into at least two. VCA of each jet was determined separately and summed up. After PMVR, measurement of one jet is exemplarily shown. VCA: vena contracta area. DMR: degenerative mitral regurgitation. MR: mitral regurgitation. Σ: two residual jets were summed up. ASE: American Society of Echocardiography

3D–colour Doppler datasets of the mitral valve were obtained from mid-oesophageal views. Seven electrocardiographically triggered sequential 3D–scans were composed for a 3D–colour full volume dataset. A post-hoc analysis was performed using commercially available software packages (Xcelera R3.2 L1, version 3.2.1.820–2011; Qlab, version 10.5, Philips Medical Systems, Amsterdam, Netherlands) according to current guidelines [2]. Tissue priority and tissue threshold were set to factory settings. All recorded 3D–colour full volume datasets were checked for lines of disagreement between neighbouring 3D subvolumes (“stitching artefacts”) within the VCA borders or “dropouts” within the dataset. Care was taken to identify blooming effects, rendering the Doppler signal larger than the laminar jet core itself [2]. If artefacts were present, the patients were excluded from further analyses (8 subjects). The median 3D frame rate came to 18 Hz, as recommended [22], with a narrow interquartile range (15 to 18 Hz; minimum 8 Hz in 1 subject). VCA was defined as the cross-sectional area of the narrowest portion of the proximal regurgitant jet through the closed mitral valve in early-to mid-systole [8, 23]. The datasets were manually cropped to provide a direct en-face view perpendicular to the jet direction. The Nyquist limit was stepwise reduced to a median of 41.1 cm/s (30.8;41.1) to visualise the colour flow regurgitant jet with maximum clarity as previously described [24, 25]. To identify the level of the narrowest portion of the regurgitant jet, the datasets were tomographically sliced. A manual planimetry of the colour Doppler signal was performed (Fig. 1). Care was taken to measure only the central laminar jet core [26] of highest, similar, transverse velocity and to exclude low-velocity eddies as recommended by previous publications and the current guidelines [2, 27]. As after PMVR there used to be multiple jets, VCA of each jet was determined separately and summed up as for complex mitral regurgitation [9].

### Statistical analysis

Categorical data are expressed as percentages. Their differences were tested for significance by Pearson’s chi-squared test. The distributions of continuous variables were assessed for normality by Shapiro-Wilk test. If normally distributed, they are expressed as mean ± standard deviation. Significance of differences was tested by Student’s t-test for dependent or independent variables, respectively. Two-way analysis of variance (two-way ANOVA) was computed to analyse the influence of categorical independent variables on left ventricular volumes and ejection fraction, respectively. When normal distribution was rejected, median and interquartile range (P25; P75) are given and variables are shown as Turkey box plots. Mann-Whitney U test, Wilcoxon signed-rank test and Friedman’s test with consecutive Dunn’s multiple comparison test were performed, as appropriate. Effect size was approximated as Cohen’s d or Hedges’s g [28] using dedicated software [29]. To assess the reduction in VCA, the ratio (VCAr) was calculated as quotient (VCAr = VCA PMVR/VCA at baseline). The absolute area of VCA reduction (VCAdiff) was defined as difference (VCAdiff = VCA baseline–VCA PMVR). VCAdiff gives the VCA reduction in absolute numbers [cm^2^]. Six-minute walk change (6MWc) was calculated as difference (6MWc = distance after–before PMVR) [30]. Kendall rank correlation coefficient (**τ)** was calculated to measure the degree of correlation of non-parametric data.

All statistical analyses were performed using SPSS statistics version 22 (IBM, Armonk, New York, USA) and GraphPad Prism Version 6.00 (GraphPad software, La Jolla, California, USA). Statistical significance was assigned at a two-sided *p*-value of less than 0.05.

### Power calculation analysis

For post-hoc power analysis G-Power [31] (version 3.1.9.2) was employed. A post-hoc power calculation was performed for the two main readouts (decrease in VCA and six-minute walking distance). It revealed sufficient power (β < 0.0001/0.01) for decrease in VCA/6 MW (Additional file 2).

## Results

### Patients’ characteristics

Baseline characteristics of the registry of the University Hospital Regensburg, Germany, are depicted in Table 1. Twenty nine patients were included for further analyses (Table 2). Patients were characterised by higher age (77.0 ± 5.8 years), advanced heart failure (75.9% in NYHA class III or IV), severely limited physical capacity in terms of short 6 MW (257.5 ± 82.5 m), high-burden of comorbidities and elevated estimated surgical risk. As depicted by Table 3, Vena contracta width (7.6 ± 1.8 mm) and effective regurgitant orifice area according to PISA method (0.45 ± 0.25 cm^2^) were measured elevated by the initial TEE. The aetiology of severe MR was considered DMR in one third (*n* = 10) and FMR in two thirds (*n* = 19) of patients. Four of 10 DMR cases were due to flail leaflet. In 7 patients, a follow-up 3D–TEE was available 41 (35;48) days after PMVR.

**Table 1:** Regensburg registry and other published trials and registries including patients treated by the MitraClip system

	Regensburg	GRASP [17]	TRAMI [19]	MitraSWISS [18]	Pilot Registry [21]^a^	ACCESS-EU [20]	EVEREST-II [1]
Year of publication		2016	2016	2014	2014	2013	2011
Participants	102	180	749	74	628	567	184
Female [%]	42.2	38.3	38.6	27	36.9	36.2	38
Age [years]	77.0 ± 5.8	71.6 ± 9.8	76 (10)^b^	72 ± 12	74.2 ± 9.7	73.7 ± 9.6	67.3 ± 12.8
MR grade III/IV [%]	100	–	93.8	100	86.1^c^	97.7	96
DMR [%]	28.4	18.3	27.8	38	22.8	20.6	74
FMR [%]	71.6	81.7	71.3	62	72.0	69.3	27
NYHA III/IV [%]	75.5	81.1	89	–	85.5	84.9	52
EuroScore II	5.1 ± 5.9	7.6 ± 6.4	–	–	–	–	–
LogEuroScore [%]	26.6 ± 18.0	–	20 (19)f	21 ± 17	20.4 ± 16.7	23.0 ± 18.3	–
Regurgitant orifice area [cm^2^]	0.40 ± 0.18	–	–	–	0.43 ± 0.16	–	0.56 ± 0.38

Shown are mean and standard deviation or proportions (if not indicated otherwise)

^a^Transcatheter Valve Treatment Sentinel Pilot Registry, ^b^median (IQR), ^c^severe (graded as mild, moderate, severe). *MR* mitral regurgitation, *DMR* degenerative mitral regurgitation, *FMR* functional mitral regurgitation, *NYHA* New York Heart Association functional classification

**Table 2:** Baseline characteristics of the study sample

Age [years]	77.0 ± 5.8
Female	41.4 (12/29)
Heart rate [bpm]	74 ± 9
Systolic blood pressure [mmHg]	119 ± 19
Diastolic blood pressure [mmHg]	67 ± 14
Body mass index [kg/m^2^]	25.8 ± 4.2
NT-proBNP [pg/ml] median(P25;75)	3618 (1619; 5782)
Serum creatinine [mg/dl] median(P25;75)	1.1 (1.0; 1.6)
logEuroScore [%] median(P25;75)	18.5 (12.7; 32.2)
NYHA functional class	
I	0 (0/29)
II	24.1 (7/29)
III	62.1 (18/29)
IV	13.8 (4/29)
Comorbidities	
DCM	10.3 (3/29)
Coronary artery disease	62.1 (18/29)
Diabetes mellitus II	34.5 (10/29)
Medical/Device treatment	
High-ceiling diuretics	100 (29/29)
ACE inhibitors	48.3 (14/29)
MRA	62.1 (18/29)
Beta-blocker	89.7 (26/29)
CRT	6.9 (2/29)

Shown are percentage of subjects (number of subjects / total number of subjects in parentheses) or mean ± standard deviation, if not indicated otherwise

*NYHA* New York Heart Association, *DCM* dilated cardiomyopathy, *ACE* angiotensin- converting enzyme, *MRA* mineralocorticoid receptor antagonist, *CRT* cardiac resynchronization therapy

**Table 3:** Mitral regurgitation in the study sample

MR aetiology	Degenerative	34.5 (10/29)
	Functional	65.5 (19/29)
MR grading	III	17.2 (5/29)
	IV	82.8 (24/29)
Vena contracta width [mm]	Degenerative	7.30 ± 1.34
	Functional	7.79 ± 2.04
ERO [cm^2^] median (P25;P75)	Degenerative	0.45 (0.33; 0.61)
	Functional	0.36 (0.27;0.60)
Number of implanted clips	1	51.7 (15/29)
	2	48.3 (14/29)

Percentage of subjects (number of subjects / total number of subjects in parentheses). *MR* mitral regurgitation, *ERO* effective regurgitant orifice calculated by the Proximal- isovelocity surface area (PISA) method

### Effect of PMVR on MR, 6 MW and NT-proBNP levels

The effects of PMVR are depicted in Table 4. MR was decreased from median grade 4 to 1 in the total study sample. The effect was also significant analysing DMR (decrease from grade 4 (3.5;4.0) to 1 (1;1.5); *p* < 0.01) and FMR separately (decrease from grade 4 (3;4) to 1 (0.5;1.5); *p* < 0.001). Largest residual MR was graded 2.5 (*n* = 1).

**Table 4:** Effect of PMVR on mitral regurgitation, 6-min walk and LV remodelling

	Before PMVR	After PMVR	*p*-value
MR grade median (P25;P75)	4 (3.5;4.0)	1 (0.5;1.5)	< 0.001
6 min walk [m]	257.5 ± 82.5	295.7 ± 96.3	< 0.01
VCA (3D) [cm^2^] median (P25;P75)	0.89 (0.65;1.33)	0.17 (0.09;0.37)	< 0.0001
NT-proBNP [pg/ml] median(P25;75)	3618 (1619;5782)	3247 (2273;4693)	0.954
LV end-diastolic volume [ml/m^2^]	85.0 ± 26.5	79.4 ± 23.2	0.17
LV end-systolic volume [ml/m^2^]	49.8 ± 22.2	44.7 ± 17.4	0.11
LV ejection fraction [%]	42.5 ± 12.5	43.6 ± 10.1	0.51

PMVR decreased mitral regurgitation and improved six-minute walking distance in a follow-up examination 4 weeks after the intervention. NT-proBNP and left-ventricular volumes and ejection fraction remained unchanged. Shown are mean ± standard deviation, if not indicated otherwise

*PMVR* percutaneous mitral valve repair, *LV* left ventricular, *MR* mitral regurgitation, *VCA (3D)* vena contracta area determined by direct planimetry in a 3D–coulor Doppler full volume

6MW was significantly improved by PMVR. The effect was particularly pronounced in patients suffering from DMR (240.4 ± 80.3 m vs. 296.1 ± 63.0 m, Cohen’s d 0.97, *p* = 0.013, *n* = 10). In FMR, effect size was smaller and slightly missed significance (266.5 ± 84.4 m, Cohen’s d = 0.47, *p* = 0.053, *n* = 19). In consequence, PMVR achieved as early as 4 weeks after the procedure a significant improvement of 6 MW with a more distinct effect for DMR.

NT-proBNP blood levels, left-ventricular volumes and ejection fraction did not show a significant change (Table 4).

### Reduction of VCA by PMVR and consistency between intra-procedural measurement and follow-up examination

**Fig. 2:**
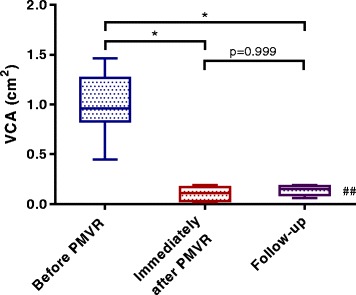
Vena contracta area remains stable 4 weeks after PMVR. Shown are values of seven patients with follow-up TEE after 4 weeks. *p < 0.05 Dunn’s multiple comparison test with ##*p* < 0.01 (Friedman test)

VCA determination was independent of age, sex, left ventricular ejection fraction, NYHA class and EuroScore. Baseline VCA was significantly reduced from 0.89cm^2^ to 0.17cm^2^ after PMVR (Table 4). Median VCAr was 0.19 (0.09;0.42). In the subgroup of patients with follow-up 3D–TEE, VCA did not vary between the measurements immediately after PMVR and during follow-up (*p* = 0.999, Fig. 2**)**. Their characteristics did not differ from the group missing a follow-up 3D–TEE examination with respect to sex, age or clinical manifestation of MR (Additional file 3).

### Correlation of intra-procedural VCA measures and grading of residual MR

**Fig. 3:**
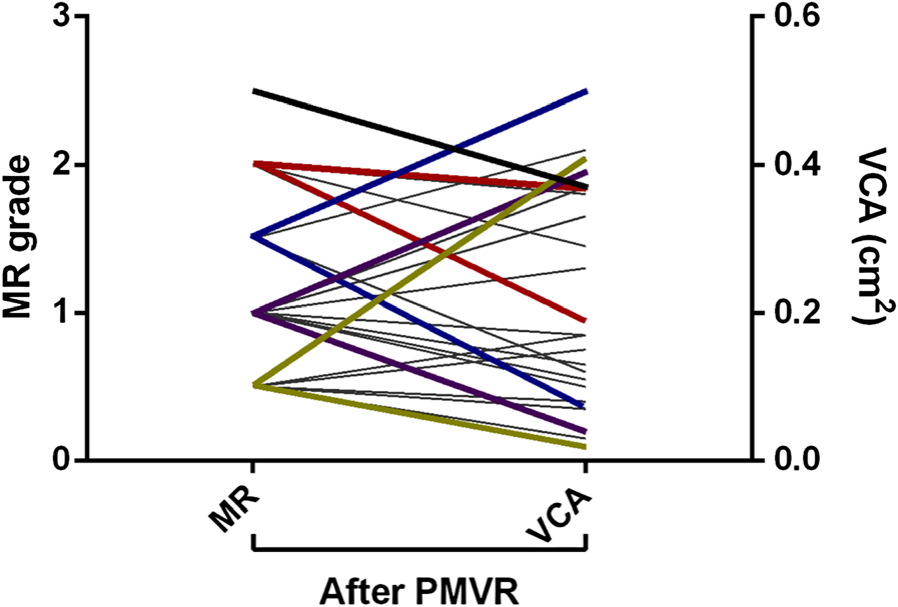
Ordinal scaled grading of residual mitral regurgitation (left y-axis) faces a wide spread of remaining vena contracta area (right y-axis). *n* = 29. Ladder plot. PMVR: percutaneous mitral valve repair. MR: mitral regurgitation. VCA: vena contracta area

Common ordinal scaled MR grading (1 to 4) and VCA were compared for evaluating residual MR. There was significant but weak correlation (τ = 0.361; *p* = 0.01). As seen in Fig. 3, the VCAs of all ordinal scaled MR grades after PMVR spread widely.

### VCA reduction as predictor of clinical outcome

**Fig. 4:**
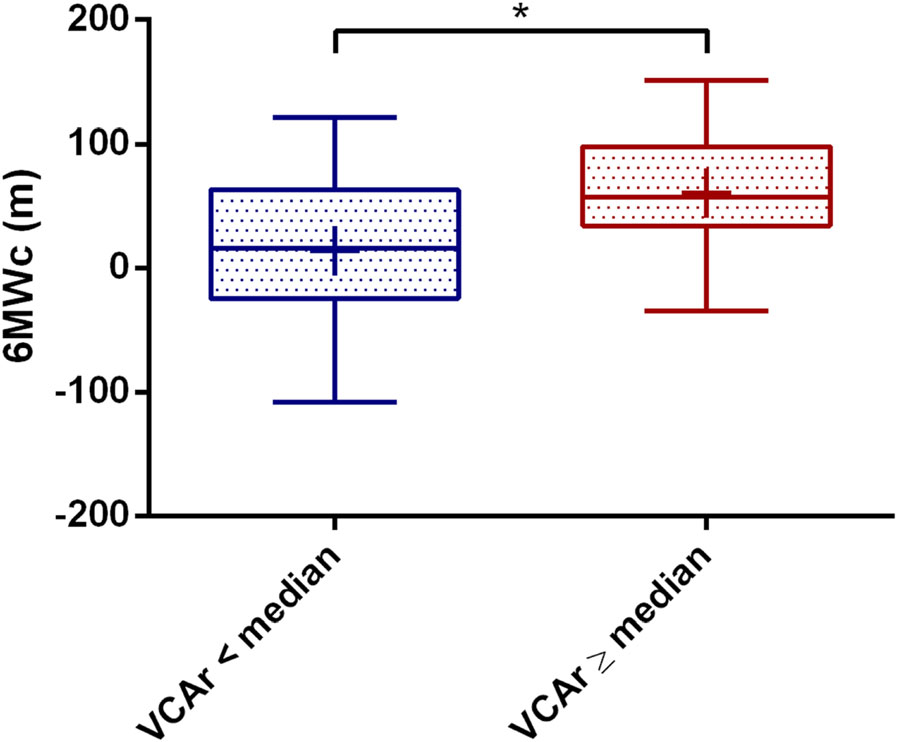
Functional success of PMVR is more pronounced, when vena contracta area is less reduced then the median of the study sample. *n* = 29. Median VCAr = 0.1868. Turkey boxplot with + marking the mean. *p < 0.05. VCAr = ratio of vena contracta area immediately after PMVR / before PMVR. 6 MWc = six-minute walk distance after PMVR – before PMVR. PMVR: percutaneous mitral valve repair

The link of MR reduction (VCAr) and an improvement of the patients’ physical capacity (6MWc) is depicted by Fig. 4. Using the median VCAr of 0.19 as cut-off value, patients with a more pronounced procedural VCA reduction, mirrored by a VCAr below the median, had a significantly smaller 6 MWc. Contrary to expectations, data imply a more modest success for functional improvement in patients, whose technical success in VCA reduction was essentially more distinct.

To scrutinize possible underlying causes, two groups divided by the median VCAr were compared. Explorative data analysis did not yield significantly differing results between groups except for the absolute area of VCA change (VCAdiff) (Additional file 4). VCAr and VCAdiff were correlated with a negative Kendall rank correlation coefficient (τ = −0.51, *p* = 0.0001). Thus, as VCAr decreases, VCAdiff increases, which seems quite conclusive.

Based on these observations, we speculated, whether effects of very large VCA could drive our results. The 75% quantile of VCAdiff was used as cut-off to differ between low and high VCAdiff (75% quantile = 1.05 cm^2^). Patients, who were suffering from FMR and exhibited a VCAdiff below the 75% quantile, had a post-procedural increase in six-minute walking distance (+43.57 ± 49.53 m, *n* = 14). By contrast, patients with larger VCAdiff came to a decreased six-minute walk distance after PMVR (−12.20 ± 76.59 m, *n* = 5). The difference showed a strong effect (Hedges’s g = −0.977) slightly missing significance (*p* = 0.078).

There was no difference between small and large VCAdiff regarding changes in post-procedural left ventricular volumes or function compared to baseline (p for end-diastolic volume/end-systolic volume/ejection fraction = 0.97/0.68/0.45, 2-way ANOVA).

Thus, in large, potentially long-standing FMR, our data was indicative for a less beneficial effect than in smaller FMR with regard to the patients’ functional outcome.

## Discussion

In this study assessing prognostic implications of VCA reduction, we observed the following key findings:

1. Patients’ six-minute walking distance was improved already 4 weeks after PMVR with a more pronounced effect for DMR.
2. VCA is significantly reduced by PMVR and its dimensions remain stable during a short-term follow-up.
3. VCA measurement immediately after PMVR improves the evaluation of small remaining MR by implying prognostic relevance for the patients’ physical capacity measured as 6MWc.

### Study sample and treatment success in the context of published literature

The baseline characteristics of the Regensburg PMVR Registry reported for the first time by our study were quite comparable to currently published results of several registries [1, 17–21] (Table 1) mirroring the real-life practice. The use in FMR was consistently reported higher than in the initial EVEREST-II trial [1]. Remarkably, in our data the effect of PMVR on patients’ functional capacity (6 MW) was more pronounced in DMR. NT-proBNP as marker of left ventricular wall stress and predictor of cardiovascular outcome [32] was elevated before and after PMVR without significant change in line with a current publication reporting no benefit of PMVR with regard to NT-proBNP levels in 144 patients [33]. Nevertheless, the field is still not settled due to contrary results in smaller samples [34]. Heterogeneous comorbidities might explain nonresponse of NT-proBNP after PMVR [35].

### VCA as independent measurement for quantifying residual MR after PMVR

In patients with high grade MR and elevated surgical risk, PMVR is increasingly used leading to significant improvements in clinical outcome [1, 14, 19]. The edge-to-edge technique of PMVR alters the complex anatomy of the mitral valve in DMR [36] and FMR [10] and creates at least two neo-orifices. The remaining MR is split into often very eccentric regurgitant jets (Fig. 1) and common parameters of echocardiographic MR assessment as width of vena contracta and effective regurgitant orifice area are not applicable. The sparse existing guidelines (e.g. of the German Cardiac Society [5], Additional file 1) recommend a multimodal approach integrating parameters determined by echocardiography (visual grading of regurgitant jet), right-heart catheterization (v-wave) and left ventriculography (regurgitant volume) with echocardiography as mainstay. Though in real-life most often used during PMVR, the qualitative as well as quantitative assessment of the regurgitant jets using colour-Doppler is imprecise and tents to an overestimation of remaining MR in the situation of multiple jets [37]. Even before the launch of PMVR, VCA was known for several strengths in the assessment of high-grade MR [8]: In an in vitro model of MR, VCA provided the strongest correlation with known orifice area (*r* = 0.92, *p* < 0.001) compared to other echocardiographic measurements, which could be translated to a prospective study comprising 61 patients with at least mild MR of different aetiology: feasibility and reproducibility was established yielding satisfying interobserver agreement (*r* = 0.96; 0.05 ± 0.02 cm^2^) [38]. In the same year, a further prospective study including 57 patients with relevant MR of different aetiologies [39] reported feasible measurements in all patients within 2.6 ± 0.7 min of measuring time and ruled out significant interobserver variability (*r* = 0.97, 0.04 ± 0.09 cm^2^). VCA is reliable in multiple jet areas, too [9].

Considering VCA measurement after PMVR, Altiok et al. set the stage by using VCA determined by 3-D-TEE to analyse the procedural effects of PMVR in FMR [11] with acceptable feasibility and reproducibility. In 2017, these data were confirmed [10] by a retrospective study comprising 97 heart failure patients with severe MR undergoing MitraClip therapy reporting adequate inter-observer variability (*r* = 0.95, *p* < 0.001). Comparing VCA to the common ordinal scale of MR grading, our data show that within each MR grade VCA still spreads. It highlights the potential of VCA measurement to increase the resolution of residual MR grading.

The intraprocedural TEE has to face the inherent problem of anaesthesia, which changes cardiac pre- and afterload influencing mitral valve function [40]. Interestingly, VCA did not change in our dataset between the intraprocedural TEE in general anaesthesia and a follow-up examination, which has been done in conscious sedation using benzodiazepines 4 weeks later. These results might indicate a quite comparability of both examinations regarding particularly VCA measurement.

### VCA as predictor of post-procedural outcome

The need for a reliable measurement of residual MR is underscored by its prognostic importance: data from the MitraSWISS registry revealed residual MR severity after PMVR as significant predictor of reduced survival after 2 years [18]. They suggested that MR should be reduced as far as possible. It has to be stressed that these analyses relied on ordinal scaled grading of residual MR by combined methods. In 2017, Alessandrini et al. measured VCA in FMR patients after PMVR. Dichotomised VCA (≥ 0.25cm^2^, upper vs. lower and middle tertile of their sample) was associated with mortality during a median follow-up of 13.4 months (HR = 3.8, CI 1.9–7.8) [10]. Our data confirm prognostic implication of remaining MR. However, since MR severity is very dynamic, we hypothesised beyond previous published analyses, that there might be differences in outcome depending on pre-procedural MR anatomy. Therefore, we wanted to assess the association of decreasing VCA (ratio post/pre-procedural) and the patients’ outcome, which was measured as gain in 6 MW. Strikingly, we observed a more pronounced increase in 6 MW 4 weeks after PMVR in patients, whose VCA was less reduced by PMVR. This paradoxical result was predominantly driven by the negative outcome of patients suffering from FMR with a large absolute difference between their VCA before and immediately after PMVR (> 75% quantile, > 1.05cm^2^). These patients lacking functional improvement after PMVR had considerably large functional regurgitant jets measured as VCA 2.5-fold higher than the cut-off value defining the edge between moderate and high-grade disease [8, 39, 41]. It is tempting to speculate whether PMVR strictly decreasing VCA might be less beneficial in very-large, presumably chronic and long-standing FMR than at an earlier point of intervention as additionally suggested by recent data [42]. For FMR comprises a variety of entities as a result of cardiac remodelling [43–46]. Thus, our study might suggest with VCA an interesting, objective, measurable pre-procedural criterion for PMVR planning. Nonetheless, this issue awaits further, strongly required prospective evaluation as patient selection in FMR for PMVR is a central and current problem and valid parameters for this purpose are strongly needed.

### Strengths and limitations

Strengths of our study include precise measurement of MR by the direct planimetry of VCA using 3D–TEE, which has already been shown to be accurate [8] and feasible in multiple jets [9] even after PMVR [10, 11]. However, data on DMR has been lacking to date. Furthermore, nor VCA neither another method has been used until now to assess prognostic implication of residual MR concerning functional treatment success. Thus, a novel approach was chosen and provided new insights. Furthermore, the recorded follow-up 3D–TEE examinations permitted an analysis of stability during short-term follow-up.

However, some limitations warrant consideration: reduction in regurgitant volume immediately after PMVR could similarly be discussed as another potential marker of later clinical outcome. Regurgitant volume is estimated as difference of stroke volumes measured at the LVOT and mitral valve level [47] or alternatively, by magnetic resonance imaging. Both methods are applicable even after MitraClip [48]. Unfortunately, we do not have this data to compare it with VCA reduction. This issue awaits future studies.

The retrospective design with a moderate-sized number of participants limits analytic options. Nevertheless, it is the first study in the field addressing this current and highly relevant issue by testing a clear and unambiguous hypothesis and using a precise measuring method as well as a quantifiable, relevant outcome variable. Thus, it allowed a thorough statistical analysis even in a medium-sized sample with appropriate statistical power.

Still, due to the small sample size, little effects can be missed and non-significant results do not rule out a potentially overseen small effect. Nonetheless, our study may report on significant effects yielded by PMVR. However, albeit significance was computed, some justified concerns about generalizability might remain because of the small sample size and should by answered by further research. To offer valid information at the moment, we provide also estimates of effect sizes beside *p*-values, which could facilitate a-priori power calculation for future prospective studies.

The study emphasised the importance of precise echocardiographic imaging in PMVR, although all large registries ignore new measurements of residual MR as VCA (Table 1). It is our hope that our results will help to design future prospective studies, which further elucidate the prognostic meaningfulness of residual MR particularly in outsized FMR.

## Conclusions

The current study confirms direct planimetry of VCA by 3D–TEE as a feasible method to quantify DMR as well as FMR in the situation of multiple neo-orifices after PMVR. PMVR reduces VCA and improves significantly 6 MWc as early as 4 weeks after the procedure with a more pronounced effect in DMR. The values of VCA determined immediately after Clip release remain stable during a short-term follow-up of 4 weeks and they imply prognostic relevance for the patients’ physical capacity measured as 6 MWc. There is some evidence that in FMR as heterogeneous disease VCA might contribute to a more substantiated estimation of treatment success.

## Additional files

Additional file 1: Recommendation for intra−/post-procedural evaluation of MR (German Cardiac Society). (DOCX 28 kb)

Additional file 2: Power calculation analysis revealed sufficient power. (DOCX 26 kb)

Additional file 3: The subgroup of patients with follow-up TEE-examination 4 weeks after PMVR does not differ from the remaining study sample concerning sex, age or clinical manifestation of MR. (DOCX 23 kb)

Additional file 4: Patients’ characteristics only differ in absolute decrease of VCA before/after PMVR between the two groups defined by median VCAr as a first hint for further insights (cf. text). (DOCX 28 kb)
