# Supplementary material for: 3D vena contracta area after MitraClip© procedure: precise quantification of residual mitral regurgitation and identification of prognostic information
Source: Cardiovasc Ultrasound. 2018 Jan 9;16:1. doi: 10.1186/s12947-017-0120-9 (PMC5759791; doi:10.1186/s12947-017-0120-9)
Supplement: Supplementary file 1 — Recommendation for intra−/post-procedural evaluation of MR (German Cardiac Society). (DOCX 28 kb) [file 12947_2017_120_MOESM1_ESM.docx]

**Additional file 1: Recommendation for intra-/post-procedural evaluation of MR (German Cardiac Society) [1]**

| **Methods** | **Measurement** | **Recommendation** |
| --- | --- | --- |
| Echocardiography  (mainstay) | visual grading of regurgitant jet (colour-Doppler) | commonly used  (mainstay) |
|  | regurgitant volume  RV=total SV-transaortic flow volume.  Total SV determined by LV volumes (Simpson or 3D); antegrade flow volume determined by LVOT-VTI (pw-Doppler) | potentially useful (after exclusion of aortic regurgitation) |
|  | vena contracta area  (determined by direct planimetry with 3D-TEE) | research |
|  | vena contracta | not recommended |
|  | ERO A (PISA method) | not recommended |
| Right heart catheterization | v-wave | potentially useful |
|  | cardiac output (thermodilution) | potentially useful |
| Left heart | regurgitant volume (ventriculography) | potentially useful |
| catheterization | “bubble” test (in combination with TEE) | potentially useful |

*RV: regurgitant volume. SV: stroke volume. LV: left ventricular. LVOT: left ventricular outflow tract. VTI: velocity time integral. pw-Doppler: pulsed-wave Doppler. (3D-)TEE: (3-dimensional) transoesophageal echocardiography. ERO A by PISA: effective regurgitant orifice area determined by the proximal isovelocity surface area method.*

**Supplementary references:**

1. Boekstegers P, Hausleiter J, Baldus S, von Bardeleben R, Beucher H, Butter C, et al. Interventionelle Behandlung der Mitralklappeninsuffizienz mit dem MitraClip®-Verfahren. Kardiologe. 2013;7:91–104.
