# Supplementary material for: 3D vena contracta area after MitraClip© procedure: precise quantification of residual mitral regurgitation and identification of prognostic information
Source: Cardiovasc Ultrasound. 2018 Jan 9;16:1. doi: 10.1186/s12947-017-0120-9 (PMC5759791; doi:10.1186/s12947-017-0120-9)
Supplement: Supplementary file 2 — Power calculation analysis revealed sufficient power. (DOCX 26 kb) [file 12947_2017_120_MOESM2_ESM.docx]

**Additional file 2: Power calculation analysis revealed sufficient power.**

A post-hoc power calculation was performed for the two main readouts (decrease in VCA and six-minute walking distance). Concerning VCA, the data reported by Altiok et al.[1] underlaid further analysis to approximate the distribution in the population: with 0.5±0.22cm^2^ (mean ± standard deviation) before and 0.21±0.13cm^2^ after PMVR an effect size of 1.51 was estimated. Setting α=0.05 and n=29 (participants in our sample), suggesting a normal response distribution and using a Wilcoxon-signed-rank test (ARE-method), a statistical power of 1.00 was calculated for our retrospective analysis to detect a difference between VCA before and after PMVR. Regarding the reduction in six-minute walking distance, an effect size of 0.92 could be computed based on the results of the MitraSWISS registry[2] with 425±104m (mean±SD) before and 523±108m after PMVR. A statistical power of 0.99 was calculated for a t-test of dependent means. Thus, the statistical power was considerably above 0.8 for both major readouts in our study sample and hence, it clearly exceeded what is commonly deemed sufficient.

As to date no data has been published estimating the effect size of the association between VCAr and 6MWc, we did not have a solid basis for power calculation regarding this issue. It is our hope that our results will help to facilitate a-prior power calculation for future prospective studies, which further elucidate the prognostic meaningfulness of VCAr in the setting of PMVR for FMR.

**Supplementary references:**

1. Altiok E, Hamada S, Brehmer K, Kuhr K, Reith S, Becker M, et al. Analysis of Procedural Effects of Percutaneous Edge-to-Edge Mitral Valve Repair by 2D and 3D Echocardiography. Circ. Cardiovasc. Imaging [Internet]. 2012;5:748–55.

2. Toggweiler S, Zuber M, Sürder D, Biaggi P, Gstrein C, Moccetti T, et al. Two-year outcomes after percutaneous mitral valve repair with the MitraClip system: durability of the procedure and predictors of outcome. Open Hear. [Internet]. 2014;1:e000056.
