# Supplementary material for: 3D vena contracta area after MitraClip© procedure: precise quantification of residual mitral regurgitation and identification of prognostic information
Source: Cardiovasc Ultrasound. 2018 Jan 9;16:1. doi: 10.1186/s12947-017-0120-9 (PMC5759791; doi:10.1186/s12947-017-0120-9)
Supplement: Supplementary file 3 — The subgroup of patients with follow-up TEE-examination 4 weeks after PMVR does not differ from the remaining study sample concerning sex, age or clinical manifestation of MR. (DOCX 23 kb) [file 12947_2017_120_MOESM3_ESM.docx]

**Additional file 3: The subgroup of patients with follow-up TEE-examination four weeks after PMVR does not differ from the remaining study sample concerning sex, age or clinical manifestation of MR. VCAr is higher in the follow-up sample.**

|  | Follow-up  (n=7) | Remaining sample  (n=22) | p-value |
| --- | --- | --- | --- |
| Sex - female | 3 | 9 | 0.93 |
| Age [years] | 80.4 ± 6.1 | 76.0 ± 5.4 | 0.08 |
| BMI [kg/m^2^] | 27.0 ± 5.5 | 25.1±3.6 | 0.31 |
| Degenerative MR | 1 | 9 | 0.20 |
| NYHA | 3 (3; 4) | 3 (2; 3) | 0.39 |
| logEuroScore | 22.1 (10.3; 34.7) | 18.5 (12.7; 31.5) | 0.98 |
| MR grade before PMVR | 4 (3; 4) | 4 (3.5; 4) | 0.26 |
| Vena contracta width [mm] | 8 (5; 9) | 7 (6; 8) | 0.75 |
| NT-proBNP [pg/ml] | 5336 (2529; 6782) | 3083 (1472; 4888) | 0.37 |

*Data are shown as absolute numbers, mean ± standard deviation, median (P25; P75), respectively. TEE: transoesophageal echocardiography. PMVR: percutaneous mitral valve repair. MR: mitral regurgitation. VC: vena contracta. VCAr: VCAratio=VCA after PMVR / VCA before PMVR. BMI: body-mass index. NYHA: New York Heart Association functional classification.*
