# Supplementary material for: 3D vena contracta area after MitraClip© procedure: precise quantification of residual mitral regurgitation and identification of prognostic information
Source: Cardiovasc Ultrasound. 2018 Jan 9;16:1. doi: 10.1186/s12947-017-0120-9 (PMC5759791; doi:10.1186/s12947-017-0120-9)
Supplement: Supplementary file 4 — Patients’ characteristics only differ in absolute decrease of VCA before/after PMVR between the two groups defined by median VCAr as a first hint for further insights (cf. text). (DOCX 28 kb) [file 12947_2017_120_MOESM4_ESM.docx]

**Additional file 4: Patients’ characteristics only differ in absolute decrease of VCA before/after PMVR between the two groups defined by median VCAr as a first hint for further insights (cf. text).**

|  | VCAr < median  (n=15) | VCAr > median  (n=14) | p-value |
| --- | --- | --- | --- |
| Sex -female | 5 | 7 | 0.36 |
| Age [years] | 78.3 ± 6.1 | 75.7 ± 5.4 | 0.24 |
| BMI [kg/m^2^] | 26.9 ± 1.4 | 24.4 ± 0.7 | 0.11 |
| Degenerative MR | 4 | 6 | 0.36 |
| NYHA | 3 (3; 3) | 3 (2; 3) | 0.57 |
| logEuroScore [%] | 22.1 (16.2; 36.7) | 16.7 (10.8; 21.7) | 0.09 |
| MR grade before PMVR | 4 (3; 4) | 4 (3.5; 4) | 0.37 |
| VCA before PMVR [cm^2^] | 1.10 ± 0.13 | 0.88 ± 0.11 | 0.18 |
| VCAdiff [cm^2^] | 0.99 ± 0.11 | 0.56 ± 0.10 | 0.01 |
| NT-proBNP [pg/ml] | 3709 (1472; 6005) | 3369 (1894; 6347) | 0.85 |
| LV end-diastolic volume [ml/m^2^] | 81.8 ± 24.7 | 88.0 ± 28.7 | 0.56 |
| LV end-systolic volume [ml/m^2^] | 47.7 ± 19.9 | 51.6 ± 24.7 | 0.66 |
| LV ejection fraction [%] | 41.7 ± 11.7 | 43.3 ± 13.6 | 0.74 |

*Data are shown as absolute numbers, mean ± standard deviation, median (P25; P75), respectively. VCA: vena contracta area. PMVR: percutaneous mitral valve repair. VCAr: ratio of vena contract area (after/before PMVR). Median VCAr: 0.1868. MR: mitral regurgitation. BMI: body-mass index. MR: mitral regurgitation. NYHA: New York Heart Association grading scheme. VCAdiff: Total difference in VCA=VCA before PMVR – VCA after PMVR. LV: left ventricular.*
